# Mother’s Milk Messaging™: trial evaluation of app and texting for breastfeeding support

**DOI:** 10.1186/s12884-022-04976-6

**Published:** 2022-08-24

**Authors:** Maya Bunik, Andrea Jimenez-Zambrano, Michael Solano, Brenda L. Beaty, Elizabeth Juarez-Colunga, Xuhong Zhang, Susan L. Moore, Sheana Bull, Jenn A. Leiferman

**Affiliations:** 1 Department of Pediatrics, University of Colorado Anschutz Medical Campus, Aurora, CO USA; 2 Adult and Child Center for Health Outcomes Research and Delivery Science, University of Colorado Anschutz Medical Campus, Aurora, CO USA; 3 Children’s Hospital Colorado, 13123 E. 16th Ave B032, Aurora, CO 80045 USA; 4 Colorado School of Public Health, University of Colorado Anschutz Medical Campus, Aurora, CO USA

**Keywords:** Breastfeeding, Breastfeeding support, Mobile/digital health, Text messaging, Perinatal care, Primary care

## Abstract

**Background:**

New mothers experience BF challenges but have limited evidence-based technology-enabled support.

**Objectives:**

1) Determine if using the Mother’s Milk Messaging™ app improved aspects of breastfeeding and breastfeeding rates and 2) Describe engagement as well as themes from the qualitative feedback on the app.

**Method:**

Randomized Controlled Trial National sample of primiparous, singleton mothers recruited online and then randomized using stratification by language into three arms: 1) BF text messages plus app; 2) BF text messages, app and physician-moderated private Facebook (FB) group; 3) Attention control group who received injury prevention texts. Exclusive breastfeeding rates as primary outcome and knowledge/attitude, confidence, and social support as secondary outcomes. We determined engagement through analysis of app usage metrics. We conducted and content-coded interviews with participants to learn more about app usage and BF experience. Due to the nature of the intervention participants could not be blinded.

**Results:**

There were a total of 346 participants in the trial, with 227 in the Intervention (*n* = 154 group 1 and *n* = 156 group 2) and 119 in the control group. Because of minimal Facebook activity, the two intervention groups 1 and 2 were combined. There were no differences in breastfeeding exclusivity and duration. (NS). Women in the intervention arm reported significantly higher confidence with breastfeeding and perceived social support to the control group (*p* < .05). Greater than 80% registered the app and those that engaged with the app had higher scores with time. Mothers appreciated receiving text messages and videos with reliable information. No harm was reported in this study.

**Conclusion:**

MMM increased confidence with breastfeeding and with gathering social supports. Exclusively BF was high in all participants. Mothers perceived it as useful and dependable especially the texting.

**Supplementary Information:**

The online version contains supplementary material available at 10.1186/s12884-022-04976-6.

## Article summary

Evaluation of Mother’s Milk Messaging™ showed no differences in breastfeeding rates but intervention mothers had higher confidence and perceived social support compared to controls.

## Introduction

Breastfeeding is the preferred method for feeding due to the benefits for both mother and infant [1–3]. Most mothers experience early breastfeeding challenges: as high as 80% of mothers report problems in the first 2–3 weeks [4–6]. Moreover, the postpartum period is a time of adjustment and recovery, and convenient breastfeeding support is desirable.

Mothers and their partners with newborns are increasingly turning to mobile health platforms and searching online. In our clinical experience providing breastfeeding support, we spend significant time debunking myths that have resulted from searching online for breastfeeding questions. Online searches lead undiscerning mothers to blog sites that do not always provide accurate or evidenced-based solutions.

While the evidence for the quality of apps to positively impact healthy behavior is growing, there is limited evidence on the effectiveness of technology-based interventions to increase breastfeeding [7–10]. Recent reports demonstrate the potential for using text messaging to increase exclusive breastfeeding, but rigorous reviews underscore the need for more well-designed scientific trials to demonstrate the benefit of using technology to promote breastfeeding [11, 12]. There are more than 2000 apps on breastfeeding, but most are assessed on the basis of feasibility, acceptability, and satisfaction, and published literature is mostly descriptive studies [13, 14]. Supplemental Nutrition Program for Women and Children pilot “Lactation Advice Through Texting Can Help (LATCH)” showed positive results in terms of acceptability and feasibility for managing access to resources, link to benefits, and appointments with peer counselors, but not breastfeeding support [15–17].

Mobile applications are becoming more popular for tracking feedings [18] (e.g., Baby Breastfeeding Tracker, MyMedela Baby Tracker), and some provide support for breastfeeding (Pacify, Breast Beginnings), but evidence-based evaluation of the mobile apps is lacking. Assessment of smartphone apps in Australia found most breastfeeding apps had minimal information with poor readability and app quality [13]. Recent systematic reviews of smartphone apps on infant feeding reported the quality as moderate in terms of engagement and functionality, but quality of information on feedings was poor [19, 20].

We developed a bilingual (Spanish and English) app Mother’s Milk Messaging™ (MMM) with daily texts to help mothers from third trimester through 3 months postpartum with peer-reviewed, evidence-based content. We evaluated MMM rigorously with mixed methods that included a randomized controlled trial, engagement analysis, and qualitative interviews.

## Methods

### Objectives

Our objectives were to 1) Determine if using the app improved aspects of breastfeeding and breastfeeding rates compared to an intention control group and 2) Describe engagement as well as themes from the qualitative feedback on the app.

### Intervention

Mothers received daily text messages for 3–4 weeks before the birth of their baby and up to 3 months after the baby’s birth. Mothers would indicate the birth by clicking a “Baby is here” button. (Supplement Screenshots of Mother’s Milk Messaging™).

MMM messages were based on two behavioral change theories. Social Cognitive Theory (SCT) operated on the premise that knowledge can be acquired by observing others in social interactions, as well as outside media influences. The Theory of Planned Behavior includes an approach that takes into account attitude, subjective norms, and perceived control of one’s behavior all together shape intentions. We also considered the established health communication approaches (i.e., Elaboration Likelihood Model of persuasion through reading messages) [21]. Over 100 text messages were initially developed by the study team and then reduced to 60 messages based on pilot testing among pregnant and postpartum women [22]. The daily messages were carefully planned during this period to be relevant to address specific issues for the breastfeeding journey.

The 20 messages delivered during pregnancy focused on increasing perceived benefits, attitudes, positive outcome expectancies, and self-efficacy related to breastfeeding. The 40 messages delivered in the postpartum period centered on strategies to garner social support and enhance behavioral skills and self-efficacy to overcome barriers to breastfeeding (e.g., latching difficulties, inadequate milk supply, return to work).

### Sample texts

- Pregnancy: Breastfeeding may be painful at first as your nipples get used to baby’s suck. A good latch & position are important to avoid problems. See our MMM Video Library for other ways to help
- First Weeks: Pop quiz! Text your answer: Colostrum (your first milk) can help A) baby poop more B) protect your baby from getting sick C) help with jaundice. D) All of the above! Follow-up text–*If you texted “D” you got it right! Colostrum is referred to as “liquid gold” because it is so valuable!*
- Later Postpartum: Good communication about bottle feeding with childcare givers is critical especially end of day timing for a breastfeeding reunion.
- Injury Prevention (Attention Control): You should wait to use a jogging stroller or bicycle trailer when your baby can hold head up well and they are at least 6 months old.

### Other app content

Information was available via short videos that were imbedded on the app and linked through YouTube. We hosted a digital story workshop with Story Center and included these more personal videos in English and Spanish. We used content from Breastfeeding Telephone Triage and Advice (Author MB) that could be found in a scrolling format by topic [23]. MMM also had a tracking feature for feedings.

### Participants

We recruited a large national sample of primiparous mothers who spoke English or Spanish (*n* = 467) through advertisements on Facebook, radio advertisements, list-serves, and local Colorado clinics from September of 2018 through January of 2019. Mothers needed to be greater than 18 years old, expecting a normal singleton birth and be at least 36 weeks along in pregnancy. They also needed to have access to a mobile phone that receives text messages and Internet (smart phone Android or iPhone).

MMM study coordinator (AJ-Z) contacted those who responded to the above, sent them a link to complete informed consent online and enroll if eligible. They were then directed to complete the baseline assessments online. Mothers were provided a $30 gift card after completion of each of the surveys at baseline, 3- and 6-month follow-up and if they participated in the qualitative interviews.

### Randomization

Mothers who consented were then randomized to one of the three arms using stratification by language. This was done to ensure that all arms had a balance of English and Spanish speaking participants. After the baseline questionnaire was completed, the participant was randomized using a block randomization scheme in REDCap to ensure balanced allocations. The research coordinator completing follow-up surveys was blind to the randomization group.

### Sample size calculation

The original study was planned with 150 women in each group. This would provide 80% (alpha = 0.05) to detect differences between the control and a given treatment group of the order of 10%. This would provide power to detect differences as those observed in the pilot study (83% vs 95%) [22].  Given that the two intervention groups were combined, this would provide 85% power to detect a difference in the order of 8–10% in the proportion of breastfeeding exclusivity depending on the proportion in the control group (e.g. a difference of 80 vs 90%).

Two intervention arms included Arm 1 Breastfeeding Text Message plus MMM APP Access, and Arm 2 Breastfeeding Text Message plus MMM APP Access plus MD Moderated Private Facebook group. Arm 3 was the attention control with injury prevention messages.

### Outcomes

The Mother’s Milk Messaging intervention aimed at improving breastfeeding outcomes. The primary outcome of the study was breastfeeding exclusivity measured at 3 and 6 months. The breastfeeding exclusivity outcome was defined as “Full and almost exclusively or exclusively fed breast milk” versus other.

Secondary outcomes related to aspects of breastfeeding were evaluated with pre-post findings using validated surveys for: Breastfeeding Intent [24, 25], Knowledge-Attitudes (Iowa Infant Feeding Attitude Scale) [26], Perceived Barriers to Breastfeeding IFPS II [27], Self-efficacy/Confidence Breastfeeding Self-Efficacy Scale Long Form (BSES) [28, 29], Perceived Social Support Assessment Tool [30, 31], Depression and Social Support Questionnaires(SSQ6) [32], and Acculturation ICR [33].

### Analyses

#### Randomized controlled trial

Baseline data were available for 467 mothers. Of those, 298 (64%) completed two follow up interviews at approximately 3 and 6 months after the due date of the child. An additional 13 (3%) had a 3-month interview only, and another 35 (7%) had a 6-month interview only. Overall, 74% of the baseline cohort had any follow-up data. We used all available data for outcomes.

The “Messages Only” group and the “Messages plus Facebook” group were combined into one intervention group, as < 3 comments were posted and only 16 participants had any views for the Facebook portion of the intervention. We made the FB group private so that outside participants could not join, encouraged anyone to post a question or comment and then we posted queries to generate discussion weekly. The average number of participants at any given time was 3–4 with only 25 ever commenting or viewing content on the FB page. Open-ended questions ranging from ‘please share your favorite nursing mother artwork to ‘tell us what surprised you the most about breastfeeding’ Comparisons between groups were done using Chi Squared or Fisher’s Exact tests for categorical variables and t-tests and Wilcoxon tests, as appropriate, for continuous variables.

Analysis of outcomes were conducted using generalized linear mixed models for binary outcomes (SAS PROC GLIMMIX) and linear mixed models for continuous outcomes (SAS PROC MIXED). We included a random intercept with unstructured covariance to account for the longitudinal nature of the data. We also performed an Intention To Treat analysis (ITT) for the entire baseline cohort with longitudinal breastfeeding exclusivity. For this ITT, all participants who had a missing value for whether they were breastfeeding exclusively at 3 and 6 months were assumed to be NOT breastfeeding exclusively. In all analyses, the independent variables were study group (Intervention/Control), Follow Up number (baseline, 1, or 2) and the interaction between the two. For the primary outcome, breastfeeding exclusivity, there were only two time points available, the first and second follow up visits. Breastfeeding outcomes were modeled as binary Yes/No. A secondary analysis of time to cessation of breastfeeding was performed using Kaplan–Meier curves and the log rank test for differences between survival functions between study groups.

#### Engagement analysis

For the analysis of engagement with the program on the outcome of breastfeeding, we tested the three-way interaction between engagement (modeled as Any vs None), study group (Intervention/Control), and follow up number. The model also included two 2-way interaction terms: study group and follow up number, and study group and engagement, as well as study group, follow-up number and measure of engagement.

#### Qualitative analysis

We conducted and content coded using ATLAS.ti qualitative interviews with participants (*n* = 66 with *n* = 29 app only, *n* = 31 pp + FB and *n* = 6 control who completed the study follow-up surveys at 3 and 6 months to learn more about app usage and their breastfeeding experience. We initially imported a list of codes [34] based on the interview guide, then we inductively generated descriptive and values codes in first-cycle coding [35]. Data were analyzed using the “editing” approach suggested by Crabtree and colleagues [36]. Code definitions were recorded in a free-standing codebook hierarchically organized by topic (i.e., family, feeding, MMM app, and power relationship) [37]. Each transcript was coded, and due to the longitudinal nature of the project, data were analyzed iteratively throughout data collection period.

This study was approved by the Colorado Institutional Review Board #10–0882. We had no reported adverse events.

## Results

### Randomized controlled trial

We compared demographics between study groups for those who completed any follow-up interview (Table 1). Since this was a national sample, with participants from 44 states. The distribution is similar when compared to the most recent US Census data.

**Table 1:** Demographics by Study Group for those who had any follow up (*n* = 346)

		**Study Group**		
**Variable**	**Category**	**Control (*N* = 119)**	**Intervention (*N* = 227)**	***P*-value**
Marital status	Single/Divorced/Widowed	16% (19)	16% (37)	0.24^b^
	Married	59% (70)	66% (150)	
	In a committed relationship but not married	25% (30)	18% (40)	
What was your method of delivery?	Vaginal	65% (71)	67% (135)	0.87^b^
	Assisted Vaginal (Forceps/vacuum)	5% (6)	4% (9)	
	Cesarean	30% (33)	28% (57)	
Consider yourself Hispanic or Latina	Yes	16% (19)	17% (39)	0.77^b^
	No	84% (100)	83% (188)	
Race	Other or prefer not to answer	17% (20)	17% (39)	0.01^b^
	Black or African American	11% (13)	3% (7)	
	White	72% (86)	80% (181)	
Health insurance	Medicaid / None / CICP	42% (50)	31% (70)	0.04^b^
	Private / Other / Military	58% (69)	69% (157)	
Highest grade or year of school completed	Attended some high school (grades 9—11)	2% (2)	3% (7)	0.88^b^
	Graduated from high school	14% (17)	14% (32)	
	Attended some college	28% (33)	24% (55)	
	Graduated from college	29% (35)	32% (73)	
	Additional school after college graduation	27% (32)	26% (60)	
Mean (SD) Age in Years		28.1 (5.2)	28.2 (5.1)	0.88^b^
Median (IQR) English Acculturation Score (*n* = 54 Hispanics)		4.0 (3.8–4.0)	4.0 (3.8–4.0)	0.92^a^
Median (IQR) Spanish Acculturation Score (*n* = 54 Hispanics)		2.8 (1.1–3.5)	2.3 (1.3–3.5)	0.76^a^
Median (IQR) Baseline Intention to Breastfeed Score		16.0 (14.0–16.0)	16.0 (14.0–16.0)	0.22^a^

^a^Wilcoxon Test

^b^Fisher’s Exact Test

The Intervention cohort had more subjects who had Private/Military/Other insurance and a larger proportion of subject who reported their race as White compared to the control group.

**Fig. 1:**
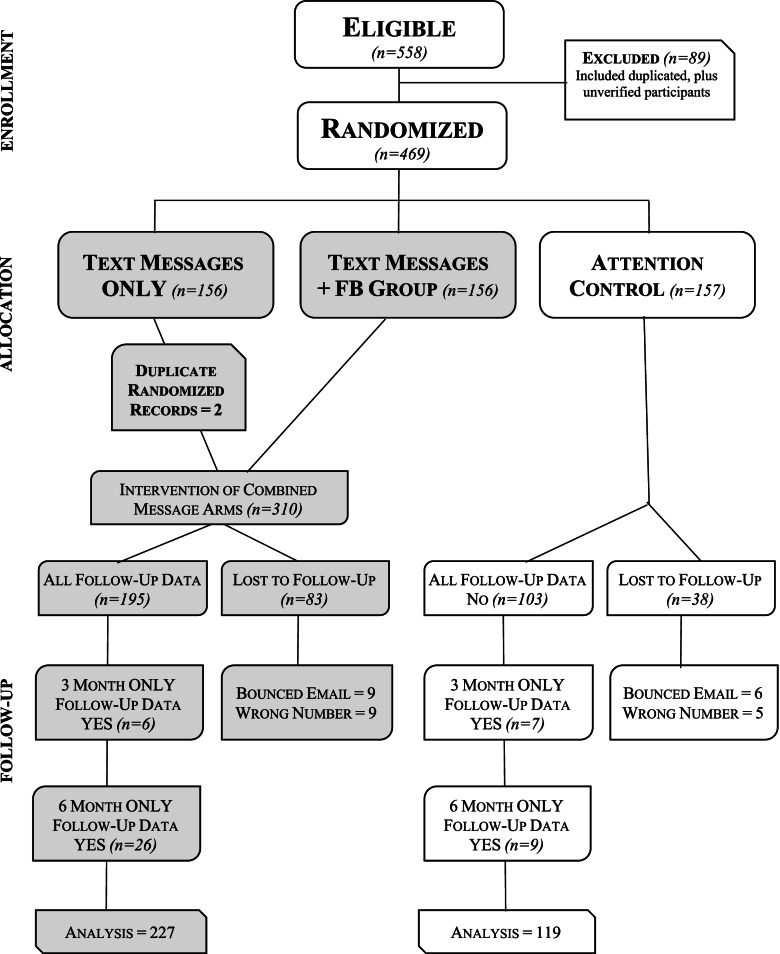
Consort Flow Diagram

The Consort Flow Diagram shows the 2 arms of the study and follow-up (Fig. 1).

The primary analysis showed no evidence of difference between the Intervention and the Control groups with regard to breastfeeding exclusivity (*p* = 0.8; Table 2). Results of the intention to treat analysis yielded consistent results with the primary analysis (*p* = 0.54; Table 2). Based on the Kaplan–Meier curves and the log rank test for time to cessation to breastfeeding, there was no evidence of a difference in the curves (*p* = 0.8).

**Table 2:** Primary outcome of exclusive breastfeeding by both study group and intention to treat for baseline cohort^a^

Primary Outcome by Study Group (*n* = 345)	Study Group		
Variable	Control (*N* = 118)	Intervention (*N* = 227)	*P*-value for interaction
Longitudinal Exclusivity (‘Full and almost exclusively or exclusively fed breast milk’			0.79
Estimated proportion (95% CI) at follow up 1	54% (42%-66%)	58% (49%-67%)	
Estimated proportion (95% CI) at follow up 2	40% (29%-53%)	47% (38%-56%)	
Intention to Treat for Baseline Cohort (*n* = 467)	Study Group		*P*-value for interaction
Variable	Control (*N* = 157)	Intervention (*N* = 310)	
Longitudinal Breastfeeding exclusivity			0.54
Estimated proportion (95% CI) at follow up 1	36% (26%-46%)	36% (29%-43%)	
Estimated proportion (95% CI) at follow up 2	26% (18%-35%)	30% (23%-37%)	

^a^Generalized linear mixed model with breastfeeding (Y/N) as the dependent variable, and time point, study group and their interaction as independent variables

Table 3 shows the secondary outcomes. There were no differences between study groups in the longitudinal outcomes of breastfeeding, Iowa Infant Feeding Attitude Scores, and Social Support Scores. However, when we looked at the Breastfeeding self-efficacy score and the Perceived breastfeeding support scale, we saw a significant interaction between the scores and study group (*p* = 0.0496 and *p* = 0.01, respectively). The intervention group had higher scores over time than the control group.

**Table 3:** Secondary outcomes by study group (*n* = 346). Models include study group, follow up number, the interaction between study group and follow up number

Variable	Study Group		*P*-value for interaction
	**Control (*N* = 118)**	**Intervention (*N* = 227)**	
Longitudinal breastfeeding			0.85
Estimated proportion (95% CI) breastfeeding at follow up 1	77% (66%-86%)	76% (67%-83%)	
Estimated proportion (95% CI) breastfeeding at follow up 2	66% (53%-77%)	66% (57%-74%)	
***Data collected at baseline, first and second follow up visits***			
**Knowledge-Attitudes (Iowa Infant Feeding Attitude Scale) score**			
*P* value for interaction term in model			0.18
Estimated Mean (95% CI) at Baseline:	67.0 (65.4–68.5)	66.2 (65.1–67.3)	
Estimated Mean (95% CI) at 3 months:	65.4 (63.8–67.0)	65.7 (64.5–66.9)	
Estimated Mean (95% CI) at 6 months:	64.9 (63.2–66.5)	65.8 (64.6–66.9)	
**Breastfeeding self-efficacy score**			
*P* value for interaction term in model			0.0496
Estimated Mean (95% CI) at Baseline:	135.3 (129.5–141.0)	129.6 (125.4–133.7)	
Estimated Mean (95% CI) at 3 months:	127.4 (121.4–133.5)	129.6 (125.1–134.0)	
Estimated Mean (95% CI) at 6 months:	126.9 (120.9–132.9)	130.0 (125.7–134.4)	
**Social Support Scale**			
*P* value for interaction term in model			0.43
Estimated Mean (95% CI) at Baseline:	33.6 (32.6 -34.6)	32.8 (32.1–33.6)	
Estimated Mean (95% CI) at 3 months:	32.1 (31.1–33.2)	32.2 (31.4–33.0)	
Estimated Mean (95% CI) at 6 months:	32.2 (31.1–33.3)	31.5 (30.8–32.3)	
**Perceived Breastfeeding Support Scale**			
*P* value for interaction term in model			0.01
Estimated Mean (95% CI) at Baseline:	11.3 (10.6–12.0)	11.1 (10.6–11.6)	
Estimated Mean (95% CI) at 3 months:	13.0 (12.3–13.7)	14.2 (13.6–14.7)	
Estimated Mean (95% CI) at 6 months:	13.4 (12.7–14.1)	13.8 (13.3–14.3)	

### Engagement

Patients who were married (*p* = 0.01), who had Private/Other/Military insurance (*p* = 0.01), higher income (*p* = 0.05), and older age (*p* = 0.02) were more likely to have engaged with the app.

Median IQR count of interactions with the app (sum of number of messages viewed, number of responses for bidirectional questions and number of videos viewed) was not significantly different between the 2 groups (*p* = 0.44).

In terms of engagement on longitudinal breastfeeding outcomes, there was no evidence of a differential effect of the intervention between those who had any engagement versus those who did not have any engagement (*p* = 0.85). We see that engagement had a positive effect on breastfeeding, (88% vs 72%) but there was no differential effect of engagement between study groups (*p* = 0.90). Results not shown.

### Qualitative results from focused interviews (Table 4)

**Table 4:** Qualitative themes and quotes

Themes	Quotes
Most participants enjoyed the app’s BF text messages as well as the texts with bidirectional quizzes	“If you’re just randomly having hard times or just need that little bit of encouragement. I think if people think you’re breastfeeding journey is going fine, they maybe aren’t outwardly expressing, “You’re doing great,” but hearing that from an app and a random text message is helpful”
	“I did actually like the updates and notifications on the app, and what to expect. That was helpful. I think it was more helpful than the lactation consultant…”
	“They were good in that, when things were difficult and I was pumping, they were kind of a reminder of the benefits of continuing to do breast milk and that I should be still continuing to try to get him to latch. So, they were kind of little reminders, without being obnoxious, that it was a good thing to do.”
	“I would say kind of having the access to the app was probably the best thing and receiving the messages.”
	“I think it’s great. The quizzes were, I don’t know, just to show– if I got something wrong, I would just look it up. I don’t know. I’s really helpful, really nice to go back to a new message every day”
Mothers appreciated the truthfulness or reliability of the information on the app	“I guess the accessibility of finding what you need without having to go throughout 50 different steps. You can go on Google and google things you’re trying to find, and then you could go and find 500 different things and none of them relate to what you're actually figuring out, but you can go on the app and find it within just a couple of minutes.”
	“I would describe it as a support program for people who want to breastfeed, where you can get answers, videos, reliable information, which is important, because you get information everywhere but knowing that it is reliable.”
	“I mean, that’s kind of how I learned. And again, when you asked about advice from other sources, there’s so many people out there who want to give you advice on how to breastfeed. There are different mommy blogs or things like that, and it was just nice to have something that allowed me to ignore that other crap and just get clear answers that I knew I could trust to be right. So, I kind of relied on it pretty heavily for that. It was like, “Okay, well, if my app doesn’t say to do this, then I'm not going to do it.” So, it was nice to be able to weed out the bad advice, I guess.”
Engagement of the app varied by each individual’s BF experiences	“I would say less [engagement with the app] just because I was pumping exclusively. Originally, I thought it would be a lot more helpful, but originally, I thought I would be naturally breastfeeding the whole time, so. I would say maybe a little less, but I still read most of the articles and messages and so forth.”
	“I think that as it got easier, I engaged less with the app. And I think that's probably why I engaged so much with it at first, and I was unsure of if what I was doing was right. And as I became more secure in what I was doing, I think I used it less.”
	“I would say both. I don't know. In the beginning, more because I was just so hyper-concerned about doing it right, and it was so important to me that I was really– any resource that I could get my hands on, I wanted. But then also sort of once I really had my feet under me, and we were on a roll, then I kind of stopped needing as much support. So that's probably when I stopped engaging as much in the app.”
	“I think it was more. If I had not been successful, I don't think I would have really used it just because it might have felt more discouraging that I couldn't do the things that the app was there for. But I think it definitely provided a very positive experience.”
	“Probably less [engagement with the app], just because there were so many difficulties upfront, and I was so exhausted, there wasn’t a lot of time to explore things.”
Most mothers engaged in the app once the baby was born even though they were entered into the study in the last 3–4 weeks of pregnancy	“I would say before I had him and probably a few weeks after is when I used it most. You're going to have a transition period right after or during birth right after that in the first few weeks until you kind of get your routine just slightly started. And then you kind of go from there, and you have yourself into a schedule for doing things.”
	“Yeah. Because I think you definitely have more questions in the beginning. It's harder in the beginning, so for the first probably 8 to 12 weeks, I would say that’s when I used it the most.”
	“I actually looked at it before, before he was born, quite a bit and in the first, I would say three to four months. And so, I think that– and I think that if it had not been helpful, I would have stopped looking at it within two weeks. But because it was so helpful, that’s why I did use it as a resource.”
	“Probably right before and in the first month afterwards.”

Four themes emerged from the 60 focused interviews with mothers who completed the 2 follow-up surveys.

- 1) Most participants enjoyed the app’s breastfeeding text messages as well as the texts with bidirectional quizzes.
- 2) Mothers appreciated the truthfulness or “reliability” (dependability) of the information on the app.
- 3) Engagement of the app varied by each individual’s breastfeeding experiences.
- 4) Most mothers engaged in the app once the baby was born.

## Discussion

Our is the first rigorous evaluation with mixed methods of a breastfeeding app. Mothers engaged with the app content and perceived MMM as useful and dependable, especially the texting feature. MMM increased confidence with breastfeeding and with gathering social supports. Most mothers breastfed with high level of exclusively, so demonstrating differences with MMM app use was more difficult. In qualitative results mothers stopped using the app if they had no problems or problems early on despite texts that recommended getting help early and often.

Eighty percent of mothers in our study downloaded the app and this is consistent with other studies in the literature well as the drop off with app engagement [38]. Satisfaction and reliability are also found in many other studies [13, 19, 39], but few were evaluated by trial and included qualitative results like ours.

We did not find any difference in breastfeeding rates between intervention and control. Breastfeeding success can be likened to any behavior change. Unless mothers are wholeheartedly intent and committed to breastfeeding, it is difficult. Demirci et al. 2018 [40] report that moms “want to do everything right”—and describe the primiparous breastfeeding experience as “fraught with internally imposed and externally reinformed pressure to produce and persevere despite inadequate breastfeeding support infrastructure.” Our qualitative analysis provided some insights on the fact that most mothers were satisfied with the daily texts and app, and it helped their breastfeeding journey. If they were having difficulties, it may have been hard to keep getting daily messages regarding breastfeeding.

Highly motivated, white married women were the highest utilizers of the MMM app despite our national online recruitment, Spanish language option, and our targeting a population that is likely seeking various apps. We were surprised that the FB group participants engaged minimally online and did not ask for help in that forum. In our pilot prior to the RCT, mothers were posting every day and at times required offline consultation. Similar to our present results, Milk Man app included a conversation forum for fathers and reported that they only posted comments online twice during the entire program [41].

Our app helped mothers with enlisting social supports and self-efficacy, and these are important antecedents to behavior change. White BK et al. 2016 [42] found engaging partners and fathers on the Milk Man app was important to breastfeeding success.

A recent trial showed improvements and satisfaction like our study but no difference in breastfeeding rates in low-income mothers [43]. Unfortunately there were confounding circumstances because 50% of the participants reported formula feeding planned after enrollment and they were excluded from the analysis [43]. Health-system sponsored apps (e.g. Circle app) have also showed change in healthy behaviors but not in health outcomes [44].

Lactapp took a retrospective look from the period of 2016–2019 and reported that baby sleep, milk extraction, breast crisis, and physiology of breastfeeding were the most common topics [45]. Apps have been successful in isolated areas such as Thailand [46] and rural parts of the US where mothers have difficulty getting breastfeeding support [47]. Telelactation support through video calls in a small study showed high breastfeeding rates [47]. Therefore, our more comprehensive app with texts, content, and videos has potential to guide new mothers on the breastfeeding journey.

Our missing data with 26% lost to follow-up is our main limitation. Our national recruitment sample was desirable but there may be less accountability when consenting online and not in person. In another app study they found that only 63% completed surveys at 3 and 6 months [48].

A recent study also has found that it hard to get mothers postpartum to fill out surveys on their phones [49]. Our recruitment sample may not be generalizable although it reflected most recent census data. We yielded motivated, white, educated, married women most of whom breastfed exclusively, but this may be indicative of the women who are using apps.

In our pre-trial pilot we found that many mothers were posting frequently and asking questions about breastfeeding, often in the middle of night. Through our qualitative interviews we learned that by the time of our actual trial mothers had other established FB groups where they were getting support. FB security issues occurred early in the study period, as did political issues that led to fear about signing up for an online study and providing personal information.

In addition, we had a possible selection bias for qualitative portion because only participants who completed both follow-up surveys of the study were eligible to participate. Therefore, the opinions might only reflect those participants who were highly engaged with our project.

## Conclusions

Mothers engaged with the app content and perceived MMM as useful and dependable, especially the texting feature. MMM increased confidence with breastfeeding and with gathering social supports. Most mothers breastfed and exclusively so demonstrating differences with MMM app use was difficult. Mothers struggling with breastfeeding early on may need more focused direction toward in-person lactation support.

## Future directions

Personalized follow-up efforts with mothers to ensure engagement and dissemination by trusted providers, home visitors or other support teams e.g., WIC in US may engage a wider range of demographics.

## Supplementary Information

**Additional file 1.** 

## Data Availability

The datasets used and/or analyzed during the current study are available from the corresponding author on reasonable request based on HIPAA requirements.

## Abbreviations

MMM: Mother’s Milk Messaging™
BF: Breastfeeding
APP: Application
PCP: Primary Care Provider

## References

[1] Joan Younger Meek, Lawrence Noble, Section on Breastfeeding; Policy Statement: Breastfeeding and the Use of Human Milk. Pediatrics. 2022;150(1):e2022057988. 10.1542/peds.2022-057988. 10.1542/peds.2022-057988 35921640

[2] Sattari M, Serwint JR, Levine DM (2019). Maternal Implications of Breastfeeding: A Review for the Internist. Am J Med.

[3] Bartick MC, Schwarz EB, Green BD (2017). Suboptimal breastfeeding in the United States: Maternal and pediatric health outcomes and costs. Matern Child Nutr.

[4] Feenstra MM, Jørgine Kirkeby M, Thygesen M, Danbjørg DB, Kronborg H. Early breastfeeding problems: A mixed method study of mothers’ experiences. Sex Reprod Healthc. 2018;16:167–74. 10.1016/j.srhc.2018.04.003 29804762

[5] Gianni ML, Bettinelli ME, Manfra P (2019). Breastfeeding Difficulties and Risk for Early Breastfeeding Cessation. Nutrients.

[6] Neifert M, Bunik M (2013). Overcoming clinical barriers to exclusive breastfeeding. Pediatr Clin North Am.

[7] Free C, Phillips G, Galli L (2013). The effectiveness of mobile-health technology-based health behaviour change or disease management interventions for health care consumers: a systematic review. PLoS Med.

[8] Wu X, Guo X, Zhang Z (2019). The Efficacy of Mobile Phone Apps for Lifestyle Modification in Diabetes: Systematic Review and Meta-Analysis. JMIR Mhealth Uhealth.

[9] Rathbone AL, Prescott J (2017). The Use of Mobile Apps and SMS Messaging as Physical and Mental Health Interventions: Systematic Review. J Med Internet Res.

[10] Flores Mateo G, Granado-Font E, Ferré-Grau C, Montaña-Carreras X (2015). Mobile Phone Apps to Promote Weight Loss and Increase Physical Activity: A Systematic Review and Meta-Analysis. J Med Internet Res.

[11] Palmer MJ, Henschke N, Bergman H (2020). Targeted client communication via mobile devices for improving maternal, neonatal, and child health. Cochrane Database Syst Rev.

[12] Unger JA, Ronen K, Perrier T (2018). Short message service communication improves exclusive breastfeeding and early postpartum contraception in a low- to middle-income country setting: a randomised trial. BJOG.

[13] Musgrave LM, Kizirian NV, Homer CSE, Gordon A (2020). Mobile Phone Apps in Australia for Improving Pregnancy Outcomes: Systematic Search on App Stores. JMIR Mhealth Uhealth.

[14] Laws RA, Denney-Wilson EA, Taki S (2018). Key Lessons and Impact of the Growing Healthy mHealth Program on Milk Feeding, Timing of Introduction of Solids, and Infant Growth: Quasi-Experimental Study. JMIR Mhealth Uhealth.

[15] Harari N, Rosenthal MS, Bozzi V (2018). Feasibility and acceptability of a text message intervention used as an adjunct tool by WIC breastfeeding peer counsellors: The LATCH pilot. Matern Child Nutr.

[16] Martinez-Brockman JL, Harari N, Segura-Pérez S, Goeschel L, Bozzi V, Pérez-Escamilla R (2018). Impact of the Lactation Advice Through Texting Can Help (LATCH) Trial on Time to First Contact and Exclusive Breastfeeding among WIC Participants. J Nutr Educ Behav.

[17] Weber SJ, Dawson D, Greene H, Hull PC (2018). Mobile Phone Apps for Low-Income Participants in a Public Health Nutrition Program for Women, Infants, and Children (WIC): Review and Analysis of Features. JMIR Mhealth Uhealth.

[18] Dienelt K, Moores CJ, Miller J, Mehta K (2020). An investigation into the use of infant feeding tracker apps by breastfeeding mothers. Health Informatics J.

[19] Almohanna AA, Win KT, Meedya S (2020). Effectiveness of Internet-Based Electronic Technology Interventions on Breastfeeding Outcomes: Systematic Review. J Med Internet Res.

[20] Taki S, Campbell KJ, Russell CG, Elliott R, Laws R, Denney-Wilson E (2015). Infant Feeding Websites and Apps: A Systematic Assessment of Quality and Content. Interact J Med Res.

[21] Karen Glanz, Barbara K. Rimer, K. Viswanath. Health Behavior and Health Education: Theory, Research and Practice. Hoboken: Wiley & Sons Inc; 2015.

[22] Maya Bunik, Jenn Leiferman, Jessica R. Ryan, Anna Furniss, Aarti Munjal, and Sheana Bull. Mother's Milk Messaging (MMM): A Pilot Study of an App to Support Breastfeeding in First Time Mothers. American Academy of Pediatrics National Conference and Exhibition Abstract, 2015. https://aap.confex.com/aap/2015/webprogrampress/Paper28725.html.

[23] Bunik M. Breastfeeding Telephone Triage and Advice. Itasca, Illinois: American Academy of Pediatrics; 2018, 2022.

[24] Nommsen-Rivers LA, Cohen RJ, Chantry CJ, Dewey KG (2010). The Infant Feeding Intentions scale demonstrates construct validity and comparability in quantifying maternal breastfeeding intentions across multiple ethnic groups. Matern Child Nutr.

[25] Nommsen-Rivers LA, Dewey KG (2009). Development and validation of the infant feeding intentions scale. Matern Child Health J.

[26] Cotelo M, Movilla-Fernández MJ, Pita-García P, Novío S (2018). Infant Feeding Attitudes and Practices of Spanish Low-Risk Expectant Women Using the IIFAS (Iowa Infant Feeding Attitude Scale). Nutrients.

[27] Hala S, Afaf M, Afnan S, Wadaa A. Breastfeeding knowledge, Attitude and Barriers among Saudi Women in Riyadh. J Nat Sci Res. 2013;3(12). ISSN 2224-3186 (Paper) ISSN 2225-0921 (Online).

[28] Dennis CL, Faux S (1999). Development and psychometric testing of the Breastfeeding Self-Efficacy Scale. Res Nurs Health.

[29] Tuthill EL, McGrath JM, Graber M, Cusson RM, Young SL (2016). Breastfeeding Self-efficacy: A Critical Review of Available Instruments. J Hum Lact.

[30] Casal CS, Lei A, Young SL, Tuthill EL (2017). A Critical Review of Instruments Measuring Breastfeeding Attitudes, Knowledge, and Social Support. J Hum Lact.

[31] Hirani SA, Karmaliani R, Christie T, Parpio Y, Rafique G (2013). Perceived Breastfeeding Support Assessment Tool (PBSAT): development and testing of psychometric properties with Pakistani urban working mothers. Midwifery.

[32] Friedman LE, Manriquez Prado AK, Santos Malavé GF (2018). Construct validity and factor structure of a Spanish-language Social Support Questionnaire during early pregnancy. Int J Womens Health.

[33] Dancel LD, Perrin E, Yin SH (2015). The relationship between acculturation and infant feeding styles in a Latino population. Obesity (Silver Spring).

[34] Miles MB, Huberman AM, Saldaña J (2014). Qualitative data analysis: a methods sourcebook.

[35] Saldaña J. The coding manual for qualitative researchers. Thousand Oaks, CA: Sage; 2012.

[36] Silverman D. Doing Qualitative Research. Thousand Oaks, California: Sage Publications; 2015.

[37] Bernard HR, Wutich AY, Ryan GW (2016). Analyzing Qualitative Data: Systematic Approaches.

[38] Krebs P, Duncan DT (2015). Health App Use Among US Mobile Phone Owners: A National Survey. JMIR Mhealth Uhealth.

[39] DeNicola N, Grossman D, Marko K (2020). Telehealth Interventions to Improve Obstetric and Gynecologic Health Outcomes: A Systematic Review. Obstet Gynecol.

[40] Demirci J, Caplan E, Murray N, Cohen S. “I Just Want to Do Everything Right:” Primiparous Women’s Accounts of Early Breastfeeding via an App-Based Diary. J Pediatr Health Care. 2018;32(2):163–72. 10.1016/j.pedhc.2017.09.010 PMC5818309 29276003

[41] White BK, Martin A, White JA (2016). Theory-Based Design and Development of a Socially Connected, Gamified Mobile App for Men About Breastfeeding (Milk Man). JMIR Mhealth Uhealth.

[42] White B, Giglia RC, White JA, Dhaliwal S, Burns SK, Scott JA (2019). Gamifying Breastfeeding for Fathers: Process Evaluation of the Milk Man Mobile App. JMIR Pediatr Parent.

[43] Lewkowitz AK, López JD, Werner EF (2021). Effect of a Novel Smartphone Application on Breastfeeding Rates Among Low-Income, First-Time Mothers Intending to Exclusively Breastfeed: Secondary Analysis of a Randomized Controlled Trial. Breastfeed Med.

[44] Cawley C, Buckenmeyer H, Jellison T, Rinaldi JB, Vartanian KB (2020). Effect of a Health System-Sponsored Mobile App on Perinatal Health Behaviors: Retrospective Cohort Study. JMIR Mhealth Uhealth.

[45] Padró-Arocas A, Quifer-Rada P, Aguilar-Camprubí L, Mena-Tudela D. Description of an mHealth tool for breastfeeding support: LactApp. Analysis of how lactating mothers seek support at critical breastfeeding points and according to their infant’s age. Res Nurs Health. 2021;44(1):173–86. 10.1002/nur.22095 33319403

[46] Wang CJ, Chaovalit P, Pongnumkul S (2018). A Breastfeed-Promoting Mobile App Intervention: Usability and Usefulness Study. JMIR Mhealth Uhealth.

[47] Kapinos K, Kotzias V, Bogen D (2019). The Use of and Experiences With Telelactation Among Rural Breastfeeding Mothers: Secondary Analysis of a Randomized Controlled Trial. J Med Internet Res.

[48] Wheaton N, Lenehan J, Amir LH (2018). Evaluation of a Breastfeeding App in Rural Australia: Prospective Cohort Study. J Hum Lact.

[49] Demirci JR, Bogen DL (2017). Feasibility and acceptability of a mobile app in an ecological momentary assessment of early breastfeeding. Matern Child Nutr.

