# Supplementary figures and images for: Mother’s Milk Messaging™: trial evaluation of app and texting for breastfeeding support
Source: BMC Pregnancy Childbirth. 2022 Aug 24;22:660. doi: 10.1186/s12884-022-04976-6 (PMC9400217; doi:10.1186/s12884-022-04976-6)

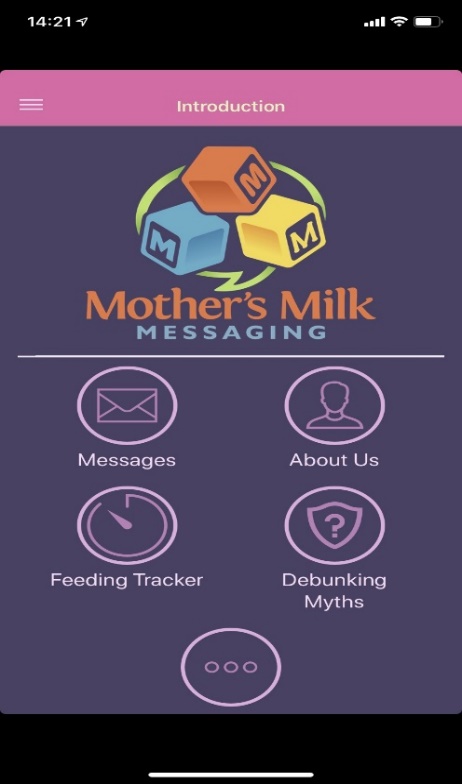

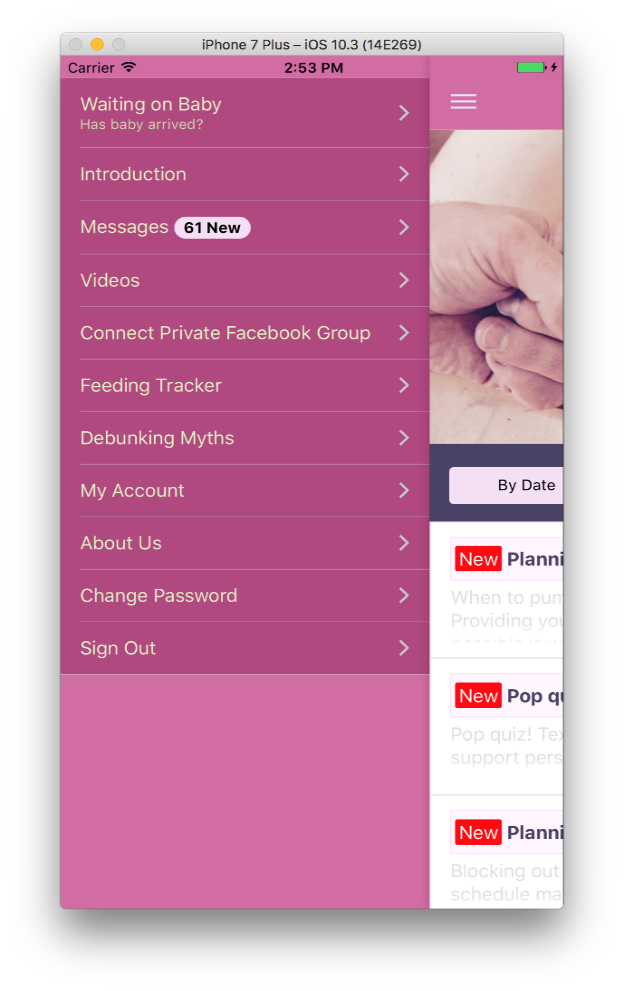

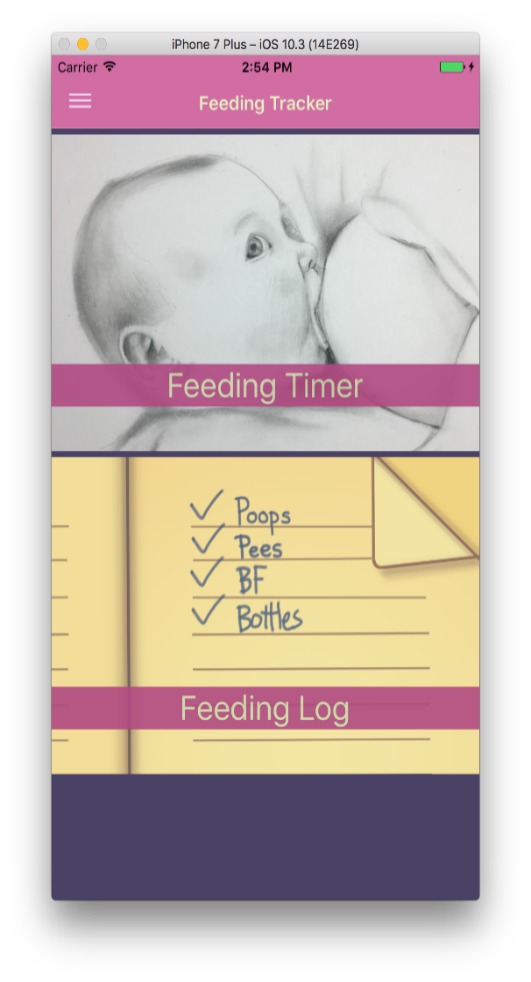

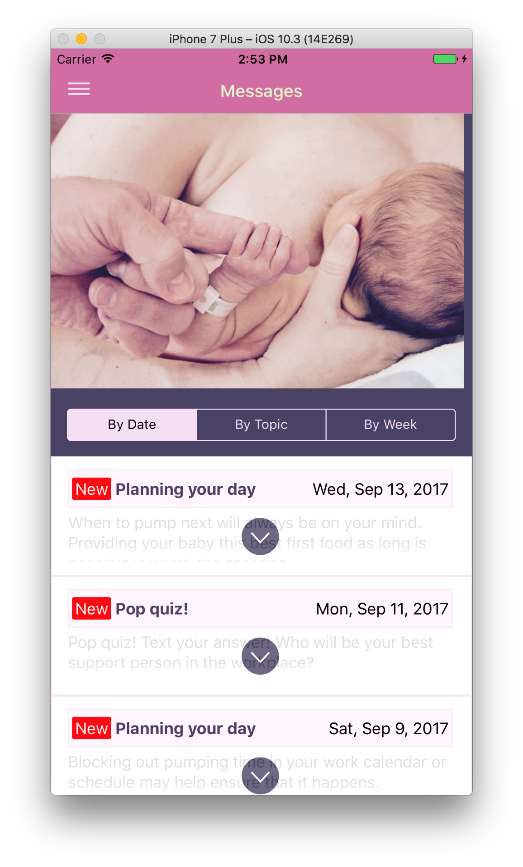


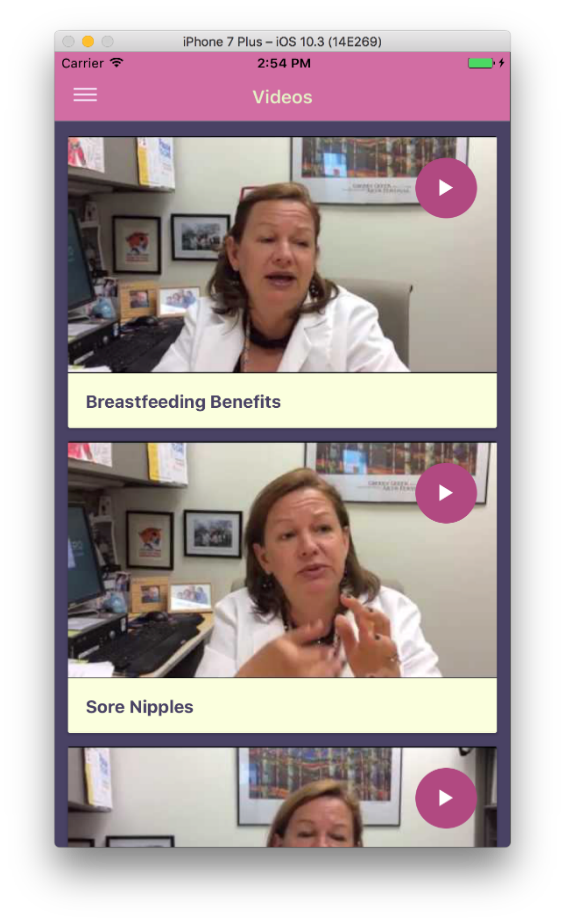

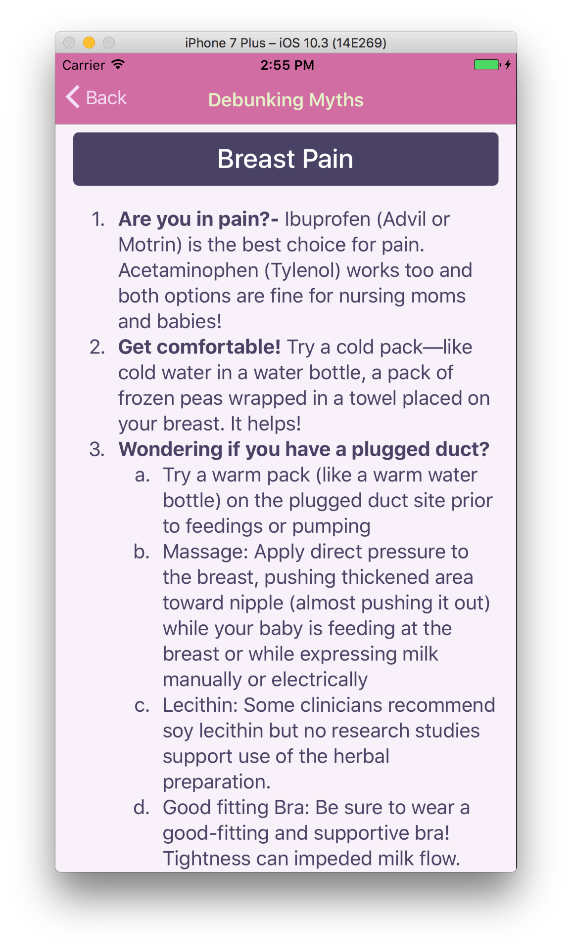

Supplement: Supplementary file 1 — Additional file 1. [file 12884_2022_4976_MOESM1_ESM.docx]
